# A Novel Approach for Multi-Lead ECG Classification Using DL-CCANet and TL-CCANet

**DOI:** 10.3390/s19143214

**Published:** 2019-07-21

**Authors:** Weiyi Yang, Yujuan Si, Di Wang, Gong Zhang

**Affiliations:** 1 College of Communication Engineering, Jilin University, Changchun 130012, China; 2 School of Electronic and Information Engineering (SEIE), Zhuhai College of Jilin University, Zhuhai 519041, China

**Keywords:** arrhythmia diagnosis, CCANet, muti-lead ECG classification, linear support vector machine, MIT-BIH database, INCART database

## Abstract

Cardiovascular disease (CVD) has become one of the most serious diseases that threaten human health. Over the past decades, over 150 million humans have died of CVDs. Hence, timely prediction of CVDs is especially important. Currently, deep learning algorithm-based CVD diagnosis methods are extensively employed, however, most such algorithms can only utilize one-lead ECGs. Hence, the potential information in other-lead ECGs was not utilized. To address this issue, we have developed novel methods for diagnosing arrhythmia. In this work, DL-CCANet and TL-CCANet are proposed to extract abstract discriminating features from dual-lead and three-lead ECGs, respectively. Then, the linear support vector machine specializing in high-dimensional features is used as the classifier model. On the MIT-BIH database, a 95.2% overall accuracy is obtained by detecting 15 types of heartbeats using DL-CCANet. On the INCART database, overall accuracies of 94.01% (II and V1 leads), 93.90% (V1 and V5 leads) and 94.07% (II and V5 leads) are achieved by detecting seven types of heartbeat using DL-CCANet, while TL-CCANet yields a higher overall accuracy of 95.52% using the above three leads. In addition, all of the above experiments are implemented using noisy ECG data. The proposed methods have potential to be applied in the clinic and mobile devices.

## 1. Introduction

Cardiovascular disease (CVD) is one of the most common causes of death, widely distributed throughout the world. According to the relevant statistical data, more than 17 million people die of cardiovascular disease each year, accounting for approximately 1/3 of the total deaths [1]. More seriously, some experts predict that the number of CVD patients is still growing, and will reach 23 million by the year 2030 [2]. Hence, it is of profound significance to timely diagnose and treat CVDs. Currently, electrocardiograms (ECGs) are widely used by medical specialists to diagnose abnormal states of the heart. However, the ECG acquisition process is inevitably affected by interference and noise. Meanwhile, the morphological changes of ECGs are not easily observable under certain disease conditions such as atrial premature beats as capturing visually subtle changes in long-term ECG signals is hard and time-consuming. To address these issues, the development of computer- aided devices for monitoring long-term ECG has been receiving increasing attention from researchers in the field.

ECG is the P-QRS-T wave. In the past few decades, ECGs have been extensively adopted as materials for arrhythmia diagnosis. Table 1 lists the related literatures, recording the methods and consequences for the detection of arrhythmia using various machine learning (ML) techniques. Among them, some researchers focus on the process for extracting ECG discriminating features, such as time domain features [3,4,5,6,7,8], frequency domain features [5,8,9,10], morphological features [3,4,5,7,8], statistical features [5,11], etc. Furthermore, others have studied classification models that can help in the analysis of ECGs representing different arrhythmias, among which the support vector machines (SVMs) [5,6,7,8,10,11] and neural networks [12,13,14,15] have been extensively employed.

Although so many methods exist for the detection of arrhythmias, it is still essential to further explore this field. Firstly, the ECGs can be recorded as different leads according to the various locations of the acquisition on the body, causing differences in the information of the heart state recorded by different multi-lead ECGs. Among these leads, limb leads (I, II, and III leads) can reflect changes in ECG from the facade of the heart, while the chest leads (V1~V6 leads) record changes of the ECGs in the cross-section of the heart. In addition, for the same type of leads (limb leads or chest leads), the change in ECG acquisition location leads to certain differences in the potential information provided by different leads. For example, the ECGs of V1 and V5 leads are all collected from the chest. However, the V1 lead contains more changes in the state of the left ventricle, while the V5 lead mainly records the potential information of the right ventricle. Hence, compared to one-lead ECGs, multi-lead ECGs can better reflect the state of the heart. Since the above methods can only process single-lead ECG signals, the other-lead ECG information reflecting the differences of cardiac states is not effectively utilized. Meanwhile, the quality of the signal collected from a certain lead may be poor, leading to possible difficulties in real-scenario arrhythmia detection using one-lead ECGs. Secondly, most of the above classification methods require the use of certain algorithms in advance to remove noise from the ECG signals [19], which may be due to the fact they can hardly deal with the noisy data. To address these issues, we propose in this paper a novel ECG recognition method. The aim of this work is to develop muti-lead ECG identification methods using a combination of canonical correlation analysis network (CCANet) [20] and linear support machine (SVM). Specifically, CCANet is an extraction method for sparse features and is produced by combining canonical correlation analysis (CCA) and cascaded convolutional network. CCANet can mine potential useful information by taking advantage of the correlation of multi-lead ECGs. In this work, the CCANet developed by Yang et al. [20] is employed for classifying dual-lead ECGs (DL-CCANet). In addition, we have developed a novel TL-CCANet for specifically handling three-lead ECG data. However, the features extracted by CCANet have high dimensionality and sparsity, making it difficult for most existing classifiers to handle such features well. Hence, a linear SVM suitable for this task is used as the classifier model in this work. To evaluate the effectiveness of them, two open databases were adopted as the experimental dataset. Among these databases, we select II lead and V1 lead for DL-CCANet, which is because that the ECGs of II lead and V1 lead have clear P waves. The P wave reflects the excitement of the atriums, while the QRS wave and the T wave reflect the excitement of the ventricles. Among these waves, the amplitude of P wave are general subtler. Therefore, to make full use of P waves, the use of II lead and the V1 lead are essential. For the TL-CCANet, we add an additional V5 lead as experimental data. Furthermore, the ECG classification results of CCANet were compared with RandNet and PCANet. The combination of PCANet and linear SVM was employed in this field in 2018 [21].

The main novel contributions of this paper may be summarized as follows:

*Dual-lead ECG classification systems.* We have designed a DL-CCANet, which consists of canonical correlation analysis (CCA), cascaded and convolutional network, binary hash encoding, block-wise histogram, linear SVM classifier.

*Three-lead ECG classification systems.* We have designed a TL-CCANet, consisting of canonical correlation analysis (CCA), cascaded and convolutional network, binary hash encoding, block-wise histogram, linear SVM classifier.

The rest of this paper is structured as follows: we firstly introduce the used materials and the proposed methods in Section 2 and Section 3, respectively. In Section 4, the experimental results and comparison are presented. Finally, the conclusions and future work are described in Section 5.

## 2. Materials

### 2.1. MIT-BIH Database

The MIT-BIH arrhythmia database [22] containing 48 sets of 2-lead (lead II and mostly V1) ECG signals with corresponding annotations was employed in our study. Each signal is a 30-min recording collected at 360 sampling frequency. In this work, all the ECG sets containing II and V1 leads downloaded from PhysioBank [23] are used for verifing the performance of the DL-CCANet.

### 2.2. INCART Database

The St. Petersburg Institute of Cardiological Techniques 12-lead arrhythmia database (INCART) downloaded from PhysioBank contains 78 sets of leads I, II, III, aVR, aVL, aVF, V1, V2, V3, V4, V5 and V6-labeled ECG recordings. Each recording was recorded with 257 Hz sampling frequency and a length of 30 min. Among these recordings, ECGs of leads II, V1 and V5 are adopted as our experimental data.

## 3. Methods

**Figure 1:**
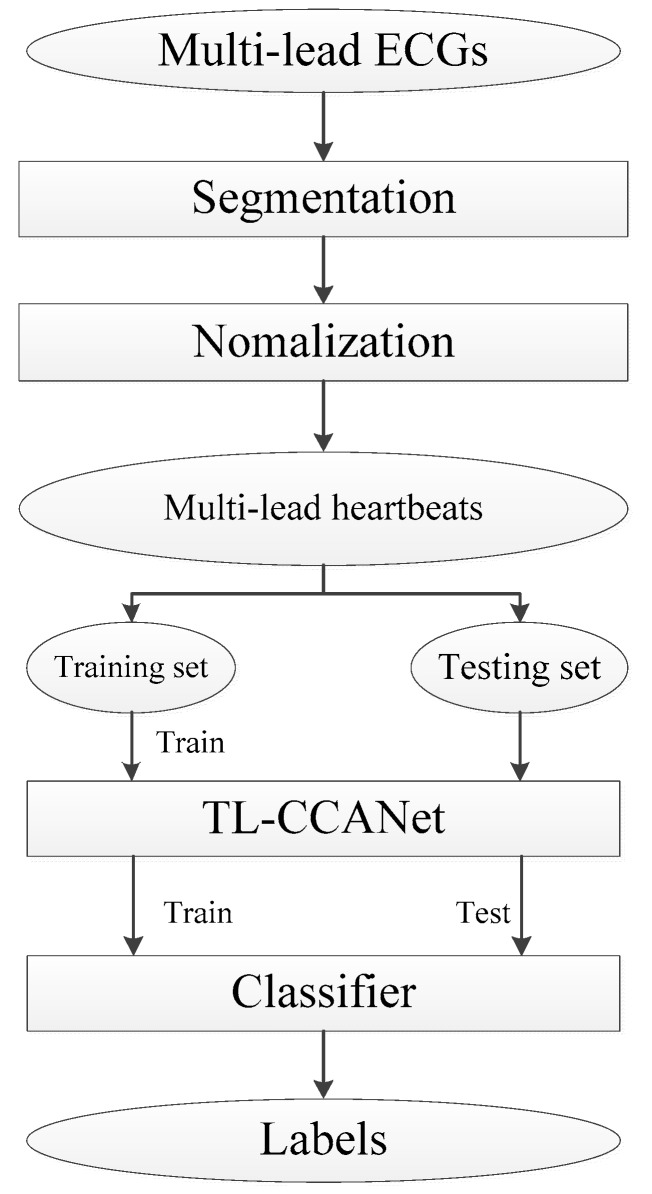
The specific process of the ECG classification based system.

In this section, we describe in detail the methods used for ECG classification. Taking TL-CCANet as an example, the specific process of the three-lead ECG classification based system is shown in Figure 1.

### 3.1. Preprocessing

In this work, we adopted R peak positions recorded in the annotations of ECG signals as the fiducial points. For each signal, we take *S*_1_ samples to the left and *S*_2_ samples to the right of R peak as a heartbeat. Next, the sample $x_{i}^{h}$ of each heartbeat is normalized as per Equation (1):

(1) $$NEW\left(x_{i}^{h}\right)\;=\frac{x_{i}^{h}-\min(x^{h})}{\max(x^{h})-\min(x^{h})}$$

where $NEW\left(x_{i}^{h}\right)$ represents the normalized *i*-th sample in a heartbeat, min(*x^h^*) and max(*x^h^*) represent samples with minimum and maximum amplitudes, respectively. The *S*_1_ and *S*_2_ for the MIT-BIH database and INCART database are shown in Table 2. The mapminmax function of MATLAB is used for normalization.

### 3.2. Feature Extraction

#### 3.2.1. DL-CCANet

**Figure 2:**
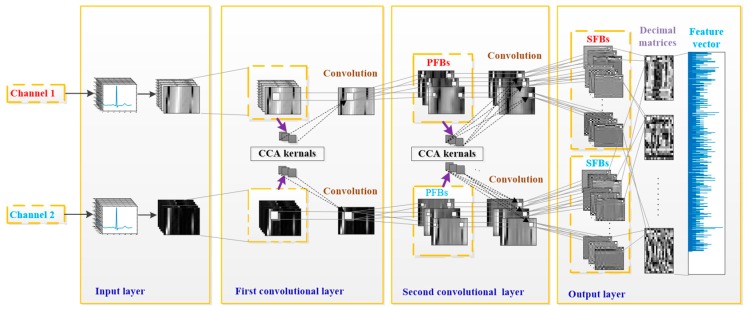
The structure of the DL-CCANet.

CCANet, designed for processing two-view images, was first proposed by Yang et al. in 2017 [20]. Compared to one-view image-based PCANet and RandNet, CCANet yields superior performance in recognizing images. A standard CCANet consists of two cascaded convolutional layers and an output layer: (1) in the convolutional layers, the CCA technique is used to extract dua-lead filter banks; (2) in the output layer, the CCA features extracted from the second convolutional layer was mapped into the final feature vector. In this work, we adopted CCANet as the feature extraction method of dual-lead ECGs. Because the 2D convolutional process in CCANet can only handle 2D matrices, an input layer is introduced before the first convolutional layer, by which the complete dual-lead CCANet (DL-CCANet) is obtained. The structure of the DL-CCANet is shown in Figure 2.

(1) Input layer

In this layer, each heartbeat containing *m* × *n* samples is reshaped into an ECG matrix $I_{i}^{h},\;i=1,2,\ldots ,N,\;h=1,2$ with a size of *m* × *n*, which represents the *i*-th heartbeat in the *h*-th ECG lead.

(2) First convolutional layer

Initial stage: Given two-lead ECG matrices $I_{i}^{1}$ and $I_{i}^{2}$, a *t*_1_ × *t*_2_ patch is adopted to extract a series of local feature blocks by centering on each pixel. We then carry out zero-center and vectorization operations on each local block, by which a set of pending vectors ${\bar{x}}_{i,1}^{h},\;{\bar{x}}_{i,2}^{h},\ldots ,{\bar{x}}_{i,mn}^{h}\in {ℜ}^{t_{1}\times t_{2}}$ are obtained. Next, the pending vectors corresponding to all the heartbeats in the *h*-th lead are concatenated into $X_{i}^{h}=\left[{\bar{x}}_{i,1}^{h},\;{\bar{x}}_{i,2}^{h},\ldots ,{\bar{x}}_{i,mn}^{h}\right]\in {ℜ}^{t_{1}t_{2}\times mn}$ and the pending matrix $X^{h}=\left[{\bar{X}}_{1}^{h},\;{\bar{X}}_{2}^{h},\ldots ,{\bar{X}}_{N}^{h}\right]\in {ℜ}^{t_{1}t_{2}\times mn}$.

Filter extraction stage: Then, the CCA filters can be obtained by reshaping several canonical vectors. With the constraints $a_{1}^{T}S_{11}a_{1}=1$ and $b_{1}^{T}S_{22}b_{1}=1$, the first canonical vector is calculated by the CCA model $\max\rho \left(a_{1},b_{1}\right)=a_{1}^{T}S_{12}b_{1},$ where $S_{ij}=\left(X^{i}\right){\left(X^{j}\right)}^{T}$, and the terms *a*_1_ and *b*_1_ are the first canonical vectors for two ECG leads, respectively. As per Equation (2), a Lagrange multiplier technique with multipliers λ and ν is adopted to solve the above CCA model:

(2) $$\theta \left(a_{1},\;b_{1}\right)=a_{1}^{T}S_{12}b_{1}-\frac{\lambda }{2}\left(a_{1}^{T}S_{11}a_{1}-1\right)-\frac{\nu }{2}\left(b_{1}^{T}S_{22}b_{1}-1\right)$$

To obtain the maximum value θ, we need to solve Equation (3).

(3) $$\left\{ \begin{array}{l} \frac{\partial \theta }{\partial a_{1}}=S_{12}b_{1}-\lambda S_{11}a_{1}=0\\ \frac{\partial \theta }{\partial b_{1}}=S_{21}a_{1}-\lambda S_{22}b_{1}=0 \end{array}\right.$$

Then, Equation (3) is converted into the forms $S_{11}^{-1}S_{12}S_{22}^{-1}S_{21}a_{1}=\lambda^{2}a_{1}$ and $S_{22}^{-1}S_{21}S_{11}^{-1}S_{12}b_{1}=\lambda^{2}b_{1}$, by which we obtain the canonical vectors *a*_1_ and *b*_1_. Next, the subsequent canonical vectors *a_l_*, *l* > 1 and *b_l_*, *l* > 1 can be calculated by solving the CCA model with the combination of Lagrange multiplier technique and new constraints $a_{i}^{T}S_{11}a_{l}=b_{i}^{T}S_{22}b_{l},\;i<l.$

As per Equation (4), all the 2*L*_1_ canonical vectors (*a_l_*, *l* = 1, 2, …, *L*_1_ and *b_l_*, *l* = 1, 2, …, *L*_1_) are mapped into matrices $V_{l}^{1},\;l=1,2,\ldots ,L_{1}$ and $V_{l}^{2},\;l=1,2,\ldots ,L_{1}$, which are adopted as the CCA filters corresponding to two ECG leads, respectively: *V* or *W*?

(4) $$\left\{ \begin{array}{l} W_{l}^{1}=mat_{k_{1},\;k_{2}}\left(a_{l}\right)\in {ℜ}^{k_{1}\times k_{2}},\;l=1,\;2,\;\cdots L_{1}\\ W_{l}^{2}=mat_{k_{1},\;k_{2}}\left(b_{l}\right)\in {ℜ}^{k_{1}\times k_{2}},\;l=1,\;2,\;\cdots L_{1} \end{array}\right.$$

where $mat_{k_{1}k_{2}}\left(•\right)$ reshapes the vectors *a_l_* and *b_l_* into matrices $W_{l}^{1}$ and $W_{l}^{2}$, and *L*_1_ is the number of CCA filters.

Convolutional stage: The preliminary feature blocks (PFBs) can be obtained as per $I_{i,l}^{h}=I_{i}^{h}*W_{l}^{h},\;l=1,2,\ldots ,L_{1},$ where ∗ is the convolutional symbol.

(3) Second convolutional layer

Initial stage: The PFBs $I_{i,l}^{h}=I_{i}^{h}*W_{l}^{h},\;l=1,2,\ldots ,L_{1}$ are employed as the input of this layer, and an initial operation similar to the previous layer is carried out to obtain $Y^{1}=\lfloor Y_{1}^{1},Y_{2}^{1},\ldots ,Y_{L_{1}}^{1}\rfloor \in {ℜ}^{k_{1}k_{2}\times L_{1}Nmn}$ and $Y^{2}=\lfloor Y_{1}^{2},Y_{2}^{2},\ldots ,Y_{L_{1}}^{2}\rfloor \in {ℜ}^{k_{1}k_{2}\times L_{1}Nmn}.$

Filter extraction stage: Similarly, taking *Y^h^*, *h* = 1,2 as the object to be processed, we employ the Lagrange multiplier technique to solve the CCA model with $S_{ij}=\left(Y^{i}\right){\left(Y^{j}\right)}^{T}$, by which the canonical vectors *c*_𝓁_ and *d*_𝓁_ can be obtained. Then, we calculate the CCA filters $V_{𝓁}^{1},\;l=1,2,\ldots ,L_{2}$ and $V_{𝓁}^{2},\;l=1,2,\ldots ,L_{2}$ as per Equation (5):

(5) $$\left\{ \begin{array}{l} V_{𝓁}^{1}=mat_{k_{1},\;k_{2}}\left(c_{𝓁}\right)\in {ℜ}^{k_{1}\times k_{2}},\;𝓁=1,\;2,\;\cdots L_{2}\\ V_{𝓁}^{2}=mat_{k_{1},\;k_{2}}\left(d_{𝓁}\right)\in {ℜ}^{k_{1}\times k_{2}},\;𝓁=1,\;2,\;\cdots L_{2} \end{array}\right.$$

where the meanings of $mat_{k_{1}k_{2}}\left(•\right)$ and *L*_2_ are the same as in Equation (4).

Convolutional stage: Similarly, the secondary feature blocks (SFBs) are calculated as per $o_{i,l}={\left\{I_{i,l}^{1}*W_{𝓁}^{1},I_{i,l}^{2}*W_{𝓁}^{2}\right\}}_{𝓁=1}^{L_{2}},\;l=1,2,\ldots ,L_{1}$, where $I_{i,l}^{1}*W_{𝓁}^{1}$ and $I_{i,l}^{2}*W_{𝓁}^{2}$ are concatenated as a whole.

(4) Output layer

In this layer, each SFB is converted into several decimal matrices as per $T_{i,l}=\sum_{𝓁}^{L_{2}}2^{𝓁-1}H\left(I_{i,l}^{1}*W_{𝓁}^{1},I_{i,l}^{2}*W_{𝓁}^{2}\right),$ where the function *H*(•) maps the $\left\lfloor I_{i,l}^{1}*W_{𝓁}^{1},I_{i,l}^{2}*W_{𝓁}^{2}\right\rfloor$ onto binary images as per Equation (6):

(6) $$H\left(c\right)=\left\{ \begin{array}{l} 1,\;c>0\\ 0,\;c<0 \end{array}\right.$$

Ultimately, the final feature vector is obtained as per *f_i_* = [*Bhist*(*T*_1_),*Bhist*(*T*_2_), …, *Bhist*($T_{L_{1}})$] $\in {ℜ}^{2^{L_{2}}L_{1}B}$, where *Bhist*(•) denotes the block segmentation (the size of the block is *u*_1_ × *u*_2_)fhg with a fixed overlap rate *R* and a histogram statistics approach, and *B* is the number of blocks collected from *T_i_*_,*j*_. The laconic workflow of the DL-CCANet is illustrated in Algorithm 1.

**Algorithm 1.** The algorithm of two convolutional stages of the DL-CCANet.
**Input:**
Raw Two-Lead Heartbeats $A_{i}^{h},\;h=1,2,\;i=1,2,\ldots ,N$

**Output:**

*f_i_*
1:Form ECG matrix $I_{i}^{h}$
2:**for** the first convolutional stage **do**
3: Form the two-lead pending matrices *X^h^*4: Compute the covariance matrix *s_ij_* of *X_i_* and *X_j_*
5: Solve the CCA model by the Lagrange multiplier technique to obtain the two-lead project directions *a*, *b*6: Construct two-lead filter banks $W_{l}^{h},\;h=1,2,\;l=1,2,\ldots ,L_{1}$
7: Calculate the preliminary feature blocks of the first convolutional stage $I_{i,l}^{h}=I_{i}^{h}*W_{l}^{h}$
8:
**end for**
9:**for** the second convolutional stage **do**10: Form the two-lead pending matrices 11: Compute the covariance matrix *s_ij_* of *Y_i_* and *Y_j_*12: Solve the CCA model to obtain the two-lead project directions *c*, *d*13: Construct two-lead filter banks $V_{𝓁}^{h},\;h=1,2,\;𝓁=1,2,\ldots ,L_{2}$
14: Calculate the output of the second convolutional stage: $o_{i,l}={\left\{I_{i,l}^{1}*W_{𝓁}^{1},I_{i,l}^{2}*W_{𝓁}^{2}\right\}}_{𝓁=1}^{L_{2}},\;l=1,2,\ldots ,L_{1}$
15:
**end for**
16: Compute the binarized images $o_{i,l}={\left\{H\left(I_{i,l}^{1}*W_{𝓁}^{1},I_{i,l}^{2}*W_{𝓁}^{2}\right)\right\}}_{𝓁}^{L_{2}}L_{1}$
17: Compute the one decimal image $T_{i,l}=\sum_{𝓁}^{L_{2}}2^{𝓁-1}H\left(I_{i,l}^{1}*W_{𝓁}^{1},I_{i,l}^{2}*W_{𝓁}^{2}\right)$
18: Construct the histogram vector *f_i_*

#### 3.2.2. TL-CCANet

**Figure 3:**
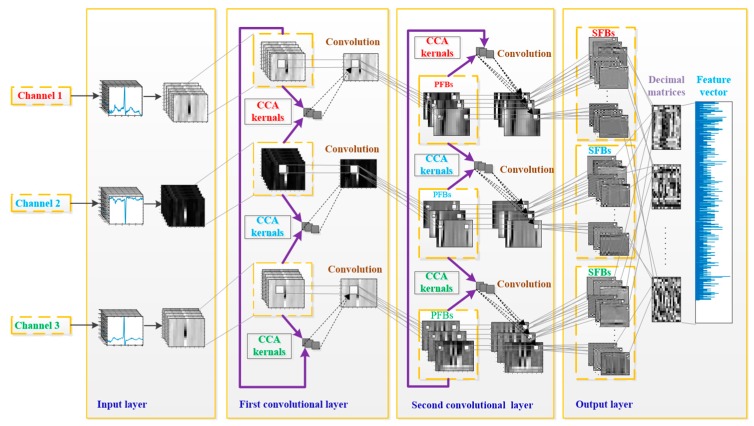
The structure of the TL-CCANet.

In this work, we developed a TL-CCANet to extract features from three-lead ECGs. TL-CCANet contains an input layer, two cascaded convolutional layers and an output layer. Different from DL-CCANet, there are three input channels for TL-CCANet, in which the CCA processing is alternately performed on the two-lead data in the two cascaded convolutional layers. Figure 3 presents the specific structure of TL-CCANet.

(1) Input layer

In this layer, ECG matrices $I_{i}^{h}$, *h* = 1, 2, 3, *h* with a size of *m* × *n* are obtained by reshaping all the heartbeats.

(2) First convolutional layer

Initial stage: The operation of this stage is the same as that in DL-CCANet, and the pending matrix $X^{h}=\lfloor {\bar{X}}_{1}^{h},{\bar{X}}_{2}^{h},\ldots ,{\bar{X}}_{N}^{h}\rfloor \in {ℜ}^{t_{1}t_{2}\times mn}$ corresponding to the *h*-th lead is then obtained.

Filter extraction stage: In this step, the CCA filters are obtained based on three combinations of *X^h^*, respectively. The specific allocation scheme is shown in Table 3.

Taking the combination 1 as an example, we first calculate the canonical vectors *a_l_* and *b_l_* by solving the CCA model between *X*^1^ and *X*^2^ using the Lagrange multiplier technique. Then, we reserve the *a_l_* corresponding to the *X*^1^, and the CCA filters are obtained as per Equation (7).

(7) $$W_{l}^{1}=mat_{k_{1},k_{2}}\left(a_{l}\right)\in {ℜ}^{k_{1}\times k_{2}},l=1,2,\cdots ,L_{1}$$

where the function $mat_{k_{1}k_{2}}\left(•\right)$ is similar to that in DL-CCANet. After processing all the above three combinations, three sets of filters $W_{l}^{1},\;W_{l}^{2}$, and $W_{l}^{3}$ are obtained.

Convolutional stage: Based on three ECG leads, we calculate the preliminary feature blocks (PFBs) according to $I_{i,l}^{h}=I_{i}^{h}*W_{l}^{h},l=1,2,\cdots ,L_{1},h=1,2,3$.

(3) Second convolutional layer

Initial stage: The $I_{i,l}^{h}=I_{i}^{h}*W_{l}^{h},l=1,2,\cdots ,L_{1}$ is employed as the input of this layer, and an operation similar to the previous layer is used to obtain $Y^{1}=\lfloor Y_{1}^{1},Y_{2}^{1},\ldots ,Y_{L_{1}}^{1}\rfloor \in {ℜ}^{k_{1}k_{2}\times L_{1}Nmn}$ and $Y^{2}=\lfloor Y_{1}^{2},Y_{2}^{2},\ldots ,Y_{L_{1}}^{2}\rfloor \in {ℜ}^{k_{1}k_{2}\times L_{1}Nmn}$.

Filter extraction stage: Taking the $Y^{1}$ and $Y^{2}$ of combination 1 in Table 4 as an example, we handle the CCA model using Lagrange multiplier technique, by which the canonical vectors $c_{𝓁}$ and $d_{𝓁}$ are obtained. Next, the CCA filters of $Y^{1}$ are obtained as per Equation (8).

(8) $$V_{l}^{2}=mat_{k_{1},\;k_{2}}\left(d_{𝓁}\right)\in {ℜ}^{k_{1}\times k_{2}},\;l=1,\;2,\;\cdots L_{1}$$

where the function $mat_{k_{1}k_{2}}\left(•\right)$ is similar to that in Equation (8). Finally, we obtain three filters $V_{l}^{1},\;V_{l}^{2}$ and $V_{l}^{3}$ for *Y*^1^, *Y*^2^ and *Y*^3^, respectively.

Convolutional stage: Similarly, the secondary feature blocks (SFBs) are calculated as per Equation (9):

(9) $$O_{i,\;l}={\left\{I_{i,\;l}^{1}*W_{𝓁}^{1},\;I_{i,\;l}^{2}*W_{𝓁}^{2},\;I_{i,\;l}^{3}*W_{𝓁}^{3}\right\}}_{𝓁=1}^{L2},\;l=1,\;2,\;\cdots ,\;L_{1}$$

(4) Output layer

Similar to that in DL-CCANet, the final feature vector *f_i_* of $I_{i}^{h}$ is obtained as per $T_{i,l}=\sum_{𝓁}^{L_{2}}2^{𝓁-1}H\left(I_{i,l}^{1}*W_{𝓁}^{1},I_{i,l}^{2}*W_{𝓁}^{2},I_{i,l}^{3}*W_{𝓁}^{3}\right)$ and *f_i_* = $\left[Bhist\left(T_{1}\right),Bhist\left(T_{2}\right),\ldots ,Bhist\left(T_{3}\right)\right]\in {ℜ}^{2^{L_{2}}L_{1}B}$. The laconic workflow of the TL-CCANet is illustrated in Algorithm 2.

The DL-CCANet and TL-CCANet are achieved by using the PCANet [24] and canoncorr function in MATLAB. Their parameters are shown in Table 5.

**Algorithm 2.** The algorithm of two convolutional stages of TL-CCANet.
**Input:**
Raw Three-Lead Heartbeats $A_{i}^{h},\;h=1,2,\;i=1,2,\ldots ,N$

**Output:**

$f_{i}$
1:Form ECG matrix $I_{i}^{h}$
2:**for** the first convolutional stage **do**
3: Form the three-lead pending matrices *X^h^*4: Compute the covariance matrix $s_{ij}^{h}$ of $X_{i}^{h}$ and $X_{j}^{h}$
5: Solve the CCA model by the Lagrange multiplier technique to obtain the three-lead project directions *a^h^, h* = 1, 2, 3 and *b^h^, h* = 1, 2, 3 6: Construct three-lead filter banks $W_{l}^{h}$, *h* = 1,2, *l* = 1, 2, …, *L*_1_7: Calculate the preliminary feature blocks of the first convolutional stage $I_{i,l}^{h}=I_{i}^{h}*W_{l}^{h}$
8:
**end for**
9:**for** the second convolutional stage **do**10: Form the three-lead pending matrices 11: Compute the covariance matrix $s_{ij}^{h}$ of $Y_{i}^{h}$ and $Y_{j}^{h}$
12: Solve the CCA model to obtain the three-lead project directions *c^h^, h* = 1, 2, 3 and *d^h^, h* = 1, 2, 3 13: Construct three-lead filter banks $V_{𝓁}^{h},\;h=1,2,3,\;𝓁=1,2,\ldots ,L_{2}$
14: Calculate the output of the second convolutional stage: $O_{i,l}={\left\{I_{i,l}^{1}*W_{𝓁}^{1},\;I_{i,l}^{2}*W_{𝓁}^{2},\;I_{i,l}^{3}*W_{𝓁}^{3}\right\}}_{𝓁=1}^{L_{2}},\;l=1,2,3,\ldots ,L_{1}$
15:
**end for**
16: Compute the binarized images $\left\{H\left(I_{i,l}^{1}*W_{𝓁}^{1},\;I_{i,l}^{2}*W_{𝓁}^{2},\;I_{i,l}^{3}*W_{𝓁}^{3}\right)\right\}$, l = 1, 2, 3, …, *L*_1_17: Compute the one decimal image $T_{i,l}=\sum_{𝓁}^{L_{2}}2^{𝓁-1}H\left(I_{i,l}^{1}*W_{𝓁}^{1},I_{i,l}^{2}*W_{𝓁}^{2},I_{i,l}^{3}*W_{𝓁}^{3}\right)$
18: Construct the histogram vector *f_i_*

### 3.3. Classification

In this work, we employ a linear support vector machine (SVM) as the classifier model. Linear support vector machines can be easily used and have a relatively simple computation process for object predictions. As we know, linear SVM makes the classification process by calculating the decision hyperplane using a linear kernel and can handle massive data efficiently and quickly. Hence, linear SVM is pretty suitable for processing the CCANet features with high dimensionality and sparsity. For a linear SVM model, error penalty factor C is a crucial parameter representing the tolerance to error. The change in the error penalty factor greatly affects the prediction results. In our experiments, we adopted the Liblinear toolkit [25] realizing linear SVM [26].

## 4. Experiments

### 4.1. Experimental Setup

The device used is a personal computer equipped with Windows 10 and 32 GB RAM, and all the experiments are implemented on Matlab 2018a using a i7-8750 CPU with a clock speed of 2.20 GHz. The proposed methodology is validated on MIT-BIH database and INCART database. In our study, we employ the DL-CCANet and the TL-CCANet to extract fusion feature from two leads and three leads, respectively. The parameters of them are shown in Table 6 and Table 7 the C of the liblinear toolkit (linear SVM). In this work, the above parameters of DL-CCANet, TL-CCANet, and linear SVM were determined through a lot of experiments on training data using changed parameters and 10-fold cross-validation (alternative 1part for validation and 9 parts for training).

To evaluate the proposed methods, we selected 15 and eight detailed categories from the MIT-BIH database and the INCART database, respectively. From the MIT-BIH database, we randomly selected 3350 heartbeats. From the INCART database, 1720 random heartbeats are used in this work. The number of each category is shown in Table 8 and Table 9, respectively.

In this work, we use k-fold cross-validation [26] to perform our experiments. Specifically, the heartbeats are divided into k equal parts. In each experiment, the alternative one part is employed as the validation data, and others are used as training data. After the above k experiments, k confusion matrices are obtained and added together. Based on the overall confusion matrix, we calculate several important indicators, which are accuracy (*Acc*), sensitivity (*Sen*), precision (*Ppv*), specificity (*Spe*), and F1-score. They can be obtained according to the following equations [15]:

(10) $$Acc=\frac{TP+TN}{TP+FP+TN+FN}\times 100\%$$

(11) $$Sen=\frac{TP}{TP+FN}\times 100\%$$

(12) $$Ppv=\frac{TP}{TP+FP}\times 100\%$$

(13) $$Spe=\frac{TN}{TN+FP}\times 100\%$$

(14) $$F1\text{-}score=\frac{2\times Ppr\times Sen}{Ppr+Sen}\times 100\%$$

where *TP*, *TN*, *FP*, and *FN* are the values of true positive, true negative, false positive, and false negative, respectively. These values of each experiment are recorded in confusion matrices.

### 4.2. Experiments on MIT-BIH Database Using DL-CCANet

The classification results for the MIT-BIH database are presented in Table 10. Overall, 95.25% of the heartbeats were correctly classified with a 4.75% error rate. Among the normal heartbeats (Nb), approximately 2.7% heartbeats of Nb class were wrongly classified as abnormal heartbeats (Ab). By contrast, for Ab, a total of 1.8% were wrongly classified as Nb class. Meanwhile, the average accuracy, Sen, Ppv, and Spe were 99.4%, 94.6%, 96.3%, and 99.6%, respectively. For the majority of the categories, over 90% sensitivity was obtained. Except for class a, the Ppv for other classes were all more than 90%. More importantly, the specificities of all the categories are over 97%, indicating that the proposed approach also has a high ability to detect negative categories. To sum up, the combination of DL-CCANet and linear SVM performed extremely well on the MIT-BIH database.

### 4.3. Experiments on INCART Database

#### 4.3.1. DL-CCANet

Table 11, Table 12 and Table 13 exhibit the classification results of DL-CCANet. These results were obtained based on various combinations of ECG leads, including combination 1 (II and V1 leads), 2 (V1 and V5 leads), and 3 (II and V5 leads). It is worth noting that the Acc and Spe for classifying each category were over 95%. In terms of Sen and Ppv, the worst result was yielded when classifying n-type heartbeats, which may be due to the minimal amount of training data with relatively insufficient classification information. In addition, over 80% of values were obtained for the precisions of all the ECG categories and the Sens of the majorities of heartbeats. From a holistic perspective, the average Accs and Spes obtained using combination 1, 2 and 3 are all over 98%, while the average Sens and Ppvs of the three schemes exceed 90% and 93%, respectively. On the whole, approximately 94% of the heartbeats were correctly classified with a 6% false detection rate. Hence, the above indicates that the combination of DL-CCANet and linear SVM can effectively identify heartbeats including N, V, A, F, n, R and j types on INCART database.

#### 4.3.2. Results Obtained Using II, V1 and V5 Leads Based TL-CCANet

Table 14 shows the confusion matrix and evaluation indicators of the classification results obtained by using II, V1, and V5 leads based TL-CCANet. Overall, 95.52% heartbeats were correctly identified with 4.48% false detection. Except for class n, over 88% Sen and 90% Ppv were obtained by classifying the remaining categories. In terms of Spe, the value is more than 98% for each abnormal heartbeats, and the average value reached 99.16%. In addition, the average Acc, average Sen, and average Ppv were 98.76%, 92.71%, and 94.88%, respectively. Compared to the DL-CCANet, TL-CCANet achieved approximately 2% higher average Sen and correctly identified more heartbeats, approximately 1.5%. More importantly, other evaluation indicators obtained by the TL-CCANet are obviously at a higher level.

### 4.4. Comparison with PCANet and RandNet

#### 4.4.1. The Comparison on MIT-BIH Database

**Figure 4:**
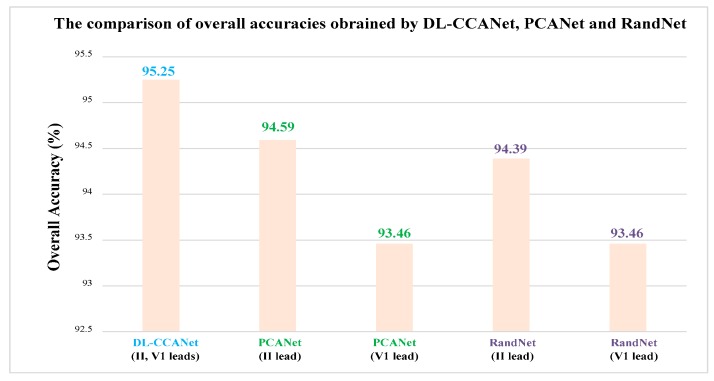
The comparison of DL-CCANet, PCANet, and RandNet on the MIT-BIH database.

**Figure 5:**
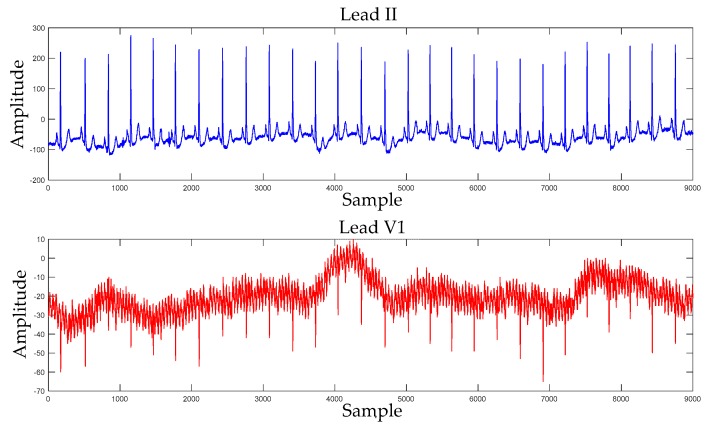
The ECG signals of lead II and V1 on the MIT-BIH database.

Figure 4 shows the comparison of overall accuracies using DL-CCANet, PCANet, and RandNet. Among these methods, PCANet and RandNet were employed to process one-lead ECGs such as lead II or lead V1. As shown in Figure 5, the lead II-based PCANet and RandNet yielded higher recognization accuracy than lead V1, which may be due to the fact that lead II and lead V1 in the MIT-BIH database were recorded with different levels of noise. The ECGs of leads II and V1 can be seen in Figure 5, where the ECGs for lead II have a clearer waveform than that for lead V1. However, the two-lead ECGs-based DL-CCANet yielded significantly better results than lead II- and lead V1- based PCANet and RandNet, indicating that DL-CCANet effectively extracts the correlation between the two leads to compensate for the lack of quality of the lead V1. Hence, the above demonstrates that DL-CCANet can solve the problem of poor quality of one-lead ECG signals.

#### 4.4.2. The Comparison on INCART Database

**Figure 6:**
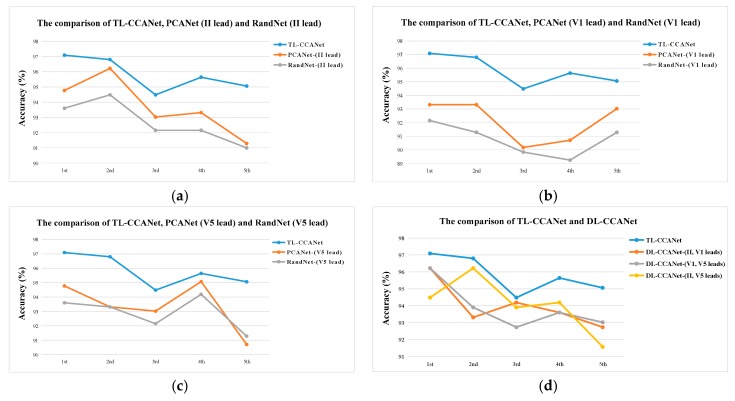
The comparison of the ECG classification performances of each fold (**a**) TL-CCANet, DL-CCANet, PCANet, and RandNet on INCART database. (**b**) TL-CCANet, PCANet(II) and RandNet(II); (**c**) TL-CCANet, PCANet(V1) and RandNet(V1); (**d**) TL-CCANet, PCANet(V5) and RandNet(V5).

**Figure 7:**
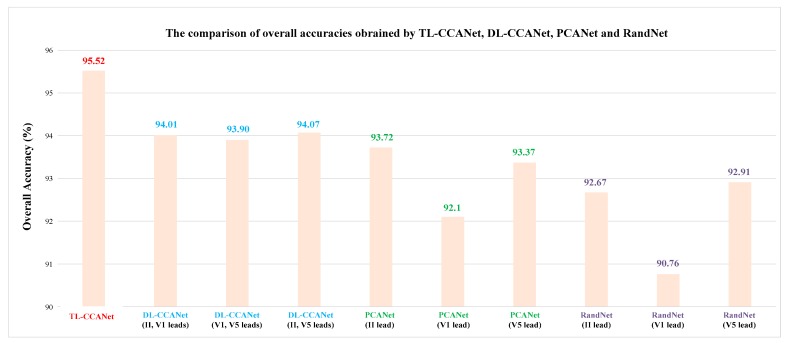
The comparison of the ECG classification performances across 10 folds using TL-CCANet, DL-CCANet, PCANet, and RandNet on INCART database.

Figure 6 and Figure 7 illustrate the ECG classification results achieved by using TL-CCANet, DL-CCANet, PCANet, and RandNet. Among them, PCANet and RandNet were used to classify heartbeats of II lead, V1 lead, and V5 lead, respectively. As per Figure 6, the line charts illustrate the recognition results when experimenting on each fold. It can be obviously seen that the TL-CCANet yielded significantly higher recognition accuracy compared to other methods.

In Figure 7, the overall accuracies of these methods across five folds are presented in the form of bar charts. Overall, the recognization accuracies were over 90% obtained by all the methods. Among them, one lead-based PCANet and RandNet have different classification results using II, V1 and V5 leads, and the recognization accuracies of V1 leads are the lowest, only 92.1% and 90.76%, respectively. Relative to one lead-based methods, DL-CCANet (II and V1 leads) and DL-CCANet (V1 and V5 leads) achieve better results (94.01% and 93.90%), indicating that the useful information of heartbeats in the V1 lead was compensated by that in the II and V5 leads. In addition, the highest recognization Accs were obtained by using three-lead (II, V1, and V5 leads) ECGs based TL-CCANet. This demonstrates that when the TL-CCANet mined relevance information of dual-lead ECGs, the useful components in third-lead ECGs were also utilized for arrhythmia diagnosis. In summary, DL-CCANet and TL-CCANet can effectively utilize the multi-lead ECGs to implement ECG classification.

**Figure 8:**
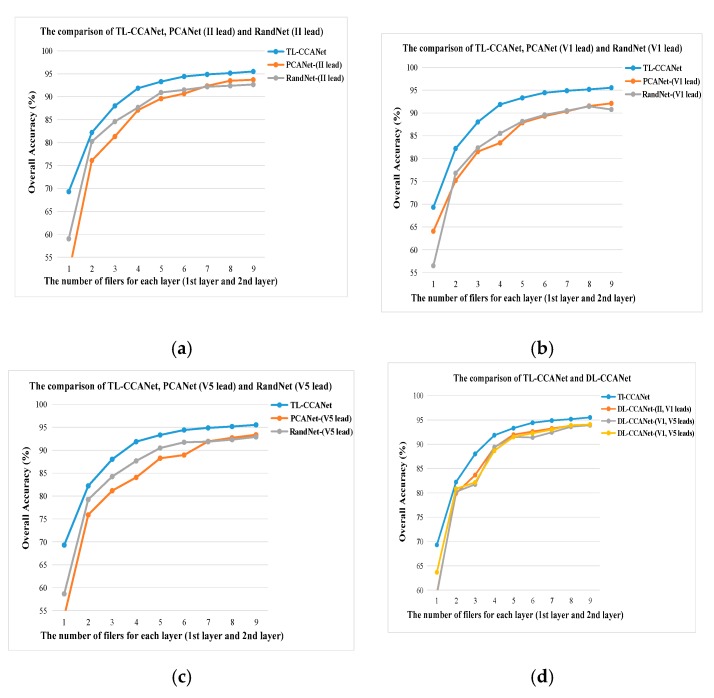
Classification results using different quantities of filters on INCART database. (**a**) TL-CCANet, PCANet (II) and RandNet (II); (**b**) TL-CCANet, PCANet (V1) and RandNet (V1); (**c**) TL-CCANet, PCANet (V5) and RandNet (V5); (**d**) TL-CCANet, DL-CCANet.

Figure 8 shows the ECG classification results obtained using different quantities of filters of DL-CCANet, TL-CCANet, PCANet, and RandNet on INCART database. Among these records, TL-CCANet achieved the best results relative to other methods when using different numbers of filters. Meanwhile, it can be observed that the influence of the number of CCA filters on the recognition effect is obvious. Specifically, as the number of filters increases, the overall recognition Acc is improved significantly and tends to be stable. However, the use of a large number of filters leads to a higher dimension of features, causing that the linear support vector machine takes longer time to implement decision making and takes up a large amount of computer memory. After a large number of experiments, 9 was selected as the optimal number of CCA filters.

### 4.5. Robustness to Noise

**Figure 9:**
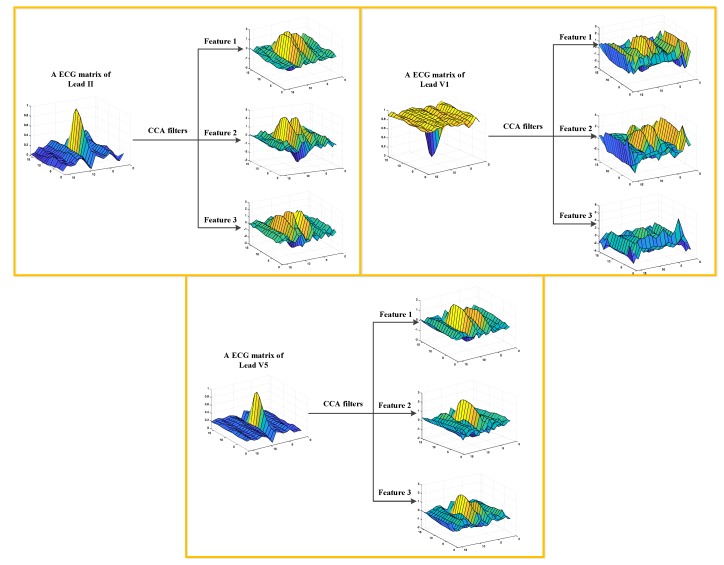
The PFBs obtained by the first convolutional layer of TL-CCANet.

As shown in Table 1, researchers have applied various methods and studied different types of arrhythmias in the related field. As we know, the ability of the proposed method to process noisy data is pretty important. In a real environment, the collected ECG signals typically contain noise with varying degrees and levels. To completely remove the noise in the signals, it is generally essential to strictly adjust the parameters of the denoising algorithm for a specific signal, resulting in a lot of wasted time and effort. Hence, the evaluation of the noise robustness of the proposed algorithm is critical. However, the majority of these methods were used to classify noise-free heartbeats, causing that the ability of these methods to deal with noisy heartbeats is not presented. In our study, the proposed method is directly applied in processing raw ECGs in the MIT-BIH database and INCART database and yields excellent performance. This may be because the process of CCA can extract correlation information between different leads. As we know, the collected ECGs mainly include baseline drift and high-frequency noise (such as EMG interference), etc. [27]. These types of noise are generally yielded by the acquisition equipment and muscle tremors. For example, baseline drift is generally caused by the displacement between the electrodes and the human body, and the degree and types of EMG are different as per the various ECG sampling positions on the body. Hence, the noise components of the different-lead ECGs have minimal correlation. As such, most of the noise information is filtered after processing the data by the CCA filters. The CCA features obtained from the first convolutional layer using the ECG matrices of II, V1, and V5 leads are illustrated in Figure 9. In addition, similar to PCANet [28], a multi-layer convolutional structure in CCANet reinforces the anti-noise and information extraction ability of CCA, supporting its application to noisy ECGs.

## 5. Conclusions

In this work, we have presented a fully automated classification method for heartbeats with various abnormal cardiac conditions. Specifically, to address some of the typical issues of previous studies, we propose two multi-lead ECGs-based feature extraction methods, which are DL-CCANet and TL-CCANet developed for extracting correlation information from dual-lead and three-lead ECGs, respectively. In the classification stage, the linear support vector machine specializing in processing high dimensional features was used as the classifier model. To verify the remarkable performance of the proposed methods, two databases from the PhysioNet PhysioBank were employed: MIT-BIH database and INCART database. By using the DL-CCANet, the highest overall accuracies of 95.25% and 94.05% were yielded in classifying detailed classes in the MIT-BIH database and INCART database, respectively. When using three-lead ECGs, the TL-CCANet achieved the highest accuracy of 95.52%, which is better than the results of DL-CCANet using two-lead ECGs in the INCART database. Meanwhile, we also used PCANet and RandNet to identify one-lead ECGs. Compared to them, the results of the proposed methods are significantly better.

Furthermore, there is an obvious difference in the quality of the signals between two leads in the MIT-BIH database. The performances of the multi-leads based methods prove that DL-CCANet effectively extracts the correlation between the two leads to compensate for the lack of quality of the lead V1. More importantly, we adopted raw ECGs as the experimental material, verifying that TL-CCANet and DL-CCANet have good ability to classify multi-lead ECGs even under noisy conditions. In terms of applications, they have minimal parameters to be adjusted and do not require the detection of P, Q, S, and T points. The proposed methods are possible to be conveniently and universally applied in the clinic or mobile devices.

The advantages of the proposed methods: (1) high overall accuracy; (2) classification of detailed classes (15 classes on MIT-BIH database and seven classes on INCART database); (3) utilization of the correlation of multi-lead ECG signals; (4) a detailed description of the methods - DL-CCANet and TL-CCANet; (5) A possible application for mobile devices; (6) a small number of parameters to be adjusted.

Among the limitations, we may mention:(1) Lower recognition performance for several classes containing minimal heartbeats such as type n in Table 10; (2) The size of ECG matrix needs to be adjusted for different databases due to the different sample rates.

In future work, we will try to solve the above limitations by utilizing more ECG leads and adopting a resampling algorithm. This work will make excellent contributions to the recognition of abnormal ECGs.

## Figures and Tables

**Table 1:** Related work of ECG classification.

Author	Year	Feature Extraction	Classifier Model	Classes	Accuracy
Park et al. [11]	2008	HOS	Hierarchical SVM	5	86%
Soria et al. [3]	2009	Morphologic and RR-intervals	Weighted LD	5	90%
Llamedo et al. [9]	2011	RR-interval and morphological features	Weighted LD	5	93%
de Lannoy et al. [4]	2012	Morphology, HOS and RR intervals	Weighted CRF	5	85%
Huang et al. [6]	2014	RR intervals and random projection	Ensemble of SVM	5	94%
Zhang et al. [7]	2014	Inter-beat and intra-beat intervals, amplitude morphology, area morphology, and morphological distance	Combined SVM	5	87%
Zhang et al. [8]	2014	Inter-beat and intra-beat intervals, amplitude morphology, area morphology, morphological distance, and wavelet coefficients	Combined SVM	5	88%
Hongqiang. [16]	2015	Combination of WPD and ApEn	SVM	5	97%
Hongqiang. [17]	2015	Combination of PCA and KICA	SVM	5	97%
Zubair et al. [12]	2016	Raw data	1D-CNN	5	93%
Acharya et al. [13]	2017	Raw data	1D-CNN	5	94%
Pławiak [10]	2018	Frequency components of the PSD of ECG signal	Evolutionary Neural System (based on single SVM)	17	90%
Pławiak [18]	2018	Frequency components of the power spectral density of the ECG signal	Genetic ensemble of SVM classifiers optimized by sets	17	91%
Yildirim et al. [14]	2018	Rescaling raw data	1D-CNN	17	91%
Pławiak [5]	2019	Frequency components of the PSD of ECG signal	Deep genetic ensemble of classifiers (DGEC)	17	95%

**Table 2:** Length of a heartbeat in MIT-BIH database and INCART database.

Database	*S*_1_	*S*_2_	Heartbeat Length (Points)	Sample Rate (Hz)
MIT-BIH database	160	200	360	360
INCART database	120	136	256	257

**Table 3:** The specific allocation results.

Lead	Combination 1	Combination 2	Combination 3
1st lead	$X^{1}$	$X^{2}$	$X^{3}$
2nd lead	$X^{2}$	$X^{3}$	$X^{1}$

**Table 4:** The specific allocation results.

Lead	Combination 1	Combination 2	Combination 3
1st lead	$Y^{3}$	$Y^{1}$	$Y^{2}$
2nd lead	$Y^{1}$	$Y^{2}$	$Y^{3}$

**Table 5:** The detailed parameters of DL-CCANet and TL-CCANet.

	Parameters	Meaning and Rang
**Input layer**	*m* × *n*	The size of the ECG matrix
	N	The number of data
**First Convolutional layer**	*t*_1_ × *t*_2_	The size of the patch (only odd integers) *t*_1_ ∊ {3:2:*m*}, *t*_2_ ∊ {3:2:*n*}
	*L*_1_	The number of filters *L*_1_ ∊ {1:1:*t*_1_*t*_2_}
**Second Convolutional layer**	*L*_2_	The number of filters *L*_2_ ∊ {1:1:*t*_1_*t*_2_}
**Output layer**	*u*_1_ × *u*_2_	The size of the block *h*_1_ ∊ {1:1:*m*}, *h*_1_ ∊ {1:1:*n*}
	*R*	The overlap ratio of the block. *R* ∊ {0.1:0.1:0.9}

**Table 6:** The parameters set for DL-CCANet and TL-CCANet.

Network Type	Layer	Value
DL-CCANet and TL-CCANet	$t_{1}\times t_{2}$	7×7
	*L*_1_	9
	*L*_2_	9
	$u_{1}\times u_{2}$	7×7
	R	0.5

**Table 7:** The parameters of liblinear toolkit.

Toolkit	The type of Solver	Penalty Factor C
Liblinear	L2-regularized L2-loss support vector classification (dual)	1

**Table 8:** The details of the categories on MIT-BIH database.

Types		Quantity
r	Rhythm change	200
N	Normal beat	1000
A	Atrial premature beat	200
V	Premature ventricular	200
P	Paced Beat	200
x	Non-conducted P-wave	100
F	Fusion of ventricular contraction	200
j	Nodal (junction) escape beat	200
L	Left bundle branch block beat	200
a	Aberrated atrial premature beat	100
J	Nodal (junction) premature beat	200
R	Right bundle branch block beat	50
!	Ventricular flutter	200
E	Ventricular escape beat	100
f	Fusion of paced and normal beat	200
Total		3350

**Table 9:** The details of the categories on INCART database.

Types		Quantity
N	Normal beat	500
V	Premature ventricular	500
A	Atrial premature beat	200
F	Fusion of ventricular contraction	200
n	Supraventricular espace beat	30
R	Right bundle branch block beat	200
j	Nodal (junction) escape beat	90
Total		1720

**Table 10:** Confusion matrix obtained using II and V5 leads across 10 folds.

		Predicted														
		r	N	A	V	P	x	F	j	L	a	J	R	!	E	f
**Original**	**r**	**190**	3	3	1	0	0	0	0	3	0	0	0	0	0	0
	**N**	2	**973**	1	2	0	0	0	19	3	0	0	0	0	0	0
	**A**	0	21	**154**	0	0	0	0	16	0	1	7	0	0	0	0
	**V**	1	8	0	**184**	0	1	2	0	1	1	0	0	2	0	0
	**P**	0	0	0	0	**200**	0	0	0	0	0	0	0	0	0	0
	**x**	1	0	0	0	0	**98**	0	0	0	0	0	0	0	1	0
	**F**	3	12	1	7	0	0	**177**	0	0	0	0	0	0	0	0
	**j**	0	7	0	0	0	0	0	**193**	0	0	0	0	0	0	0
	**L**	0	0	0	1	0	0	0	0	**199**	0	0	0	0	0	0
	**a**	1	7	1	3	0	0	1	0	0	**87**	0	0	0	0	0
	**J**	0	0	5	1	0	0	0	0	0	0	**194**	0	0	0	0
	**R**	0	1	0	0	0	0	0	1	0	0	0	**48**	0	0	0
	**!**	0	0	0	2	0	0	0	0	0	0	0	0	**198**	0	0
	**E**	0	1	0	2	0	0	0	0	0	0	0	0	1	**96**	0
	**f**	0	0	0	0	0	0	0	0	0	0	0	0	0	0	**200**
**Acc (%)**		99.5	97.4	98.3	99	100	99.9	99.2	98.7	99.8	99.9	99.6	99.9	99.9	99.9	100
**Sen (%)**		95	97.3	77	92	100	98	88.5	96.5	99.5	87	97	96	99	96	100
**Ppv (%)**		96	94.2	93.3	90.6	100	99	98.3	84.3	96.6	97.8	96.5	100	98.5	99	100
**Spe (%)**		99.8	97.5	99.7	99.4	100	100	99.9	98.9	99.8	99.9	99.8	100	99.9	100	100
**Average Acc (%)**		**99.4**														
**Average Sen (%)**		**94.6**														
**Average Ppv (%)**		**96.3**														
**Average Spe (%)**		**99.6**														
**OA (%):**		**95.3**														
**Training time (s)**		**195**														
**Classification time for single heartbeat (s)**		**0.021**														

**Table 11:** Confusion matrix obtained using II and V1 leads across 5 folds.

		Predicted							Acc (%)	Sen (%)	Ppv (%)	Spe (%)
		N	V	A	F	n	R	j				
**Original**	**N**	**484**	1	1	8	4	0	2	95.81	96.8	89.13	95.16
	**V**	0	**492**	0	8	0	0	0	98.31	98.4	95.91	98.28
	**A**	26	1	**173**	0	0	0	0	98.37	86.5	99.43	99.93
	**F**	24	17	0	**159**	0	0	0	96.67	79.5	90.86	98.95
	**n**	6	0	0	0	**24**	0	0	99.42	80	85.71	99.76
	**R**	0	1	0	0	0	**199**	0	99.94	99.5	100	100
	**j**	3	1	0	0	0	0	**86**	99.65	95.56	97.73	99.88
**Average**									**98.31**	**90.89**	**94.11**	**98.85**
**Overall accuracy (%)**									**94.01**			
**Training time (s)**									**68.8**			
**Classification time for single heartbeat (s)**									**0.017**			

**Table 12:** Confusion matrix obtained using V1 and V5 leads across 5 folds.

		Predicted							Acc (%)	Sen (%)	Ppv (%)	Spe (%)
		N	V	A	F	n	R	j				
**Original**	**N**	**487**	2	0	5	4	0	2	95.93	97.4	89.52	95.33
	**V**	3	**485**	0	10	0	2	0	97.97	97	96.04	98.36
	**A**	20	2	**174**	4	0	0	0	98.43	87	99.43	99.93
	**F**	25	14	1	**160**	0	0	0	96.57	80	89.39	98.75
	**n**	7	0	0	0	**23**	0	0	99.36	77	85.19	99.76
	**R**	0	2	0	0	0	**198**	0	99.77	99	99	99.87
	**j**	2	0	0	0	0	0	**88**	99.77	97.78	97.78	99.88
**Average**									**98.26**	**90.74**	**93.76**	**98.84**
**Overall accuracy (%)**									**93.9**			
**Training time (s)**									**72.9**			
**Classification time for single heartbeat (s)**									**0.017**			

**Table 13:** Confusion matrix obtained using II and V5 leads across 5 folds.

		Predicted							Acc (%)	Sen (%)	Ppv (%)	Spe (%)
		N	V	A	F	n	R	j				
**Original**	**N**	**487**	2	0	5	4	0	2	95.99	97.4	89.69	95.41
	**V**	4	**484**	0	11	0	1	0	97.79	96.8	95.65	98.20
	**A**	22	2	**176**	0	0	0	0	98.55	88	99.44	99.93
	**F**	17	16	1	**166**	0	0	0	97.09	83	91.21	98.95
	**n**	8	0	0	0	**22**	0	0	99.30	73.33	84.62	99.76
	**R**	1	2	0	0	0	**197**	0	99.77	98.5	99.49	99.93
	**j**	4	0	0	0	0	0	**86**	99.65	95.56	97.93	99.88
**Average**									**98.31**	**90.38**	**94**	**98.87**
**Overall accuracy (%)**									**94.07**			
**Training time (s)**									**70.2**			
**Classification time for single heartbeat (s)**									**0.017**			

**Table 14:** Confusion matrix obtained using II, V1, and V5 leads across 5 folds.

		Predicted							Acc (%)	Sen (%)	Ppv (%)	Spe (%)
		N	V	A	F	n	R	j				
**Original**	**N**	**489**	2	1	2	4	0	2	97.21	97.8	92.97	96.97
	**V**	0	490	0	10	0	0	0	98.73	98	96.65	98.61
	**A**	16	3	**179**	2	0	0	0	98.66	89.5	98.90	99.87
	**F**	14	9	1	**176**	0	0	0	97.73	88	92.15	99.01
	**n**	6	0	0	0	**24**	0	0	99.42	80	85.71	99.76
	**R**	0	2	0	0	0	**198**	0	99.88	99	100	100
	**j**	1	1	0	1	0	0	**87**	99.71	96.67	97.75	99.88
**Average**									**98.76**	**92.71**	**94.88**	**99.16**
**Overall Accuracy (%)**									**95.52**			
**Training time (s):**									**81.14**			
**Classification time for single heartbeat (s):**									**0.021**			

## References

[1] AHA Heart Disease, Stroke and Research Statistics at-Aglance. http://www.heart.org/idc/groups/ahamah-public/%40wcm/%40sop/%40smd/documents/downloadable/ucm_480086.pdf.

[2] WHO (2014). Global Status Report on Noncommunicable Diseases. http://apps.who.int/iris/bitstream/10665/148114/1/9789241564854_eng.pdf?ua=1.

[3] Soria M.L., Martínez J.P. Analysis of multidomain features for ECG classification. Proceedings of the 2009 36th Annual Computers in Cardiology Conference (CinC).

[4] De Lannoy G., François D., Delbeke J., Verleysen M. (2011). Weighted conditional random fields for supervised interpatient heartbeat classification. IEEE Trans. Biomed. Eng..

[5] Plawiak P., Acharya U.R. (2018). Novel deep genetic ensemble of classifiers for arrhythmia detection using ECG signals. Neural Comput. Appl..

[6] Huang H., Liu J., Zhu Q., Wang R., Hu G. (2014). A new hierarchical method for inter-patient heartbeat classification using random projections and RR intervals. Biomed. Eng. Online.

[7] Zhang Z., Dong J., Luo X., Choi K.S., Wu X. (2014). Heartbeat classification using disease-specific feature selection. Comput. Biol. Med..

[8] Zhang Z., Luo X. (2014). Heartbeat classification using decision level fusion. Biomed. Eng. Lett..

[9] Llamedo M., Martínez J.P. (2010). Heartbeat classification using feature selection driven by database generalization criteria. IEEE Trans. Biomed. Eng..

[10] Pławiak P. (2018). Novel methodology of cardiac health recognition based on ECG signals and evolutionary-neural system. Expert Syst. Appl..

[11] Park K.S., Cho B.H., Lee D.H., Song S.H., Lee J.S., Chee Y.J., Kim S.I. Hierarchical support vector machine based heartbeat classification using higher order statistics and hermite basis function. Proceedings of the 2008 Computers in Cardiology.

[12] Zubair M., Kim J., Yoon C. An automated ECG beat classification system using convolutional neural networks. Proceedings of the 2016 6th International Conference on IT Convergence and Security (ICITCS).

[13] Acharya U.R., Oh S.L., Hagiwara Y., Tan J.H., Adam M., Gertych A., Tan R.S., San T.R. (2017). A deep convolutional neural network model to classify heartbeats. Comput. Biol. Med..

[14] Yıldırım Ö., Pławiak P., Tan R.S., Acharya U.R. (2018). Arrhythmia detection using deep convolutional neural network with long duration ECG signals. Comput. Biol. Med..

[15] Li H., Yuan D., Ma X., Cui D., Cao L. (2017). Genetic algorithm for the optimization of features and neural networks in ECG signals classification. Sci. Rep..

[16] Li H., Feng X., Cao L., Li E., Liang H., Chen X. (2016). A new ECG signal classification based on wpd and apen feature extraction. Circ. Syst. Signal Process..

[17] Li H., Liang H., Miao C., Cao L., Feng X., Tang C., Li E. (2016). Novel ECG signal classification based on Kica nonlinear feature extraction. Circuits Syst. Signal Process..

[18] Pławiak P. (2018). Novel genetic ensembles of classifiers applied to myocardium dysfunction recognition based on ECG signals. Swarm Evol. Comput..

[19] Jin Z., Dong A., Shu M., Wang Y. (2019). Sparse ECG Denoising with Generalized Minimax Concave Penalty. Sensors.

[20] Yang X., Liu W., Tao D., Cheng J. (2017). Canonical correlation analysis networks for two-view image recognition. Inf. Sci..

[21] Yang W., Si Y., Wang D., Guo B. (2018). Automatic recognition of arrhythmia based on principal component analysis network and linear support vector machine. Comput. Biol. Med..

[22] Moody G.B., Mark R.G. (2001). The impact of the MIT-BIH arrhythmia database. IEEE Eng. Med. Biol. Mag..

[23] Goldberger A.L., Amaral L.A.N., Glass L., Hausdorff J.M., Ivanov P.C., Mark R.G., Mietus J.E., Moody G.B., Peng C.-K., Stanley H.E. (2000). PhysioBank, PhysioToolkit, and PhysioNet. Circulation.

[24] PCANet Code. https://download.csdn.net/download/txg198955/8046351.

[25] Fan R.E., Chang K.W., Hsieh C.J., Wang X.R., Lin C.J. (2008). Liblinear: A library for large linear classifification. J. Mach. Learn. Res..

[26] Kuncheva L.I. (2004). Combining Pattern Classifiers: Methods and Algorithms.

[27] Li H., Wang X., Chen L., Li E. (2014). Denoising and R-peak detection of electrocardiogram signal based on EMD and improved approximate envelope. Circuits Syst. Signal Process..

[28] Lee J.N., Byeon Y.H., Pan S.B., Kwak K.C. (2018). An EigenECG Network Approach Based on PCANet for Personal Identification from ECG Signal. Sensors.

